# The history of written word in Croatian nursing

**DOI:** 10.3325/cmj.2012.53.631

**Published:** 2012-12

**Authors:** Sonja Kalauz, Snježana Čukljek, Irena Kovačević

**Affiliations:** University of Applied Health Studies, Zagreb, Croatia

## Abstract

Today the development of a unique professional language and publishing of professional and scientific publications is the basis of every profession, including the nursing profession. The task of the unique language specific to the nursing profession is to describe the nursing profession (to make it more familiar to the other team members and clients/customers), improve communication between nurses and other team members, help in health care improvement and administration, enable comparison of health care results, improve health care outcomes, as well as facilitate health care documentation and encourage research related to nursing. From the historical point of view, the development of nursing practice in Croatia was not accompanied by professional writings until the end of the 20th century, especially not by professional articles written by nurses themselves. By analyzing old writings and handbooks, the historical development of the written word of nurses is reconstructed for the first time in the region.

## Beginnings of written word in Croatian nursing

Nursing is generally described as a very developed profession with well-organized professional associations; it is oriented toward providing services within its own autonomous field of work and it has its own independent regulations and legislation. Furthermore, as every other profession, nursing possesses systematized knowledge and skills transferable by teaching, a well-organized educational and testing system for the future members, as well as a unique specialist/professional language. The task of the language unique to nursing profession is to describe the nursing practice (make it more familiar to the other team members and clients/customers), improve communication between nurses and other team members, help in health care improvement and administration, enable comparison of health care results, improve health care outcomes, facilitate health care documentation and encourage research related to nursing. At the same time, it enables that articles, handbooks, and manuals, books, and course books are written in the native tongue of a country, with contents different from that of other scientific disciplines, which will provide a clear framework of the occupational area of the nursing profession.

It is difficult to precisely reconstruct the development of the written word in Croatian nursing. It should be pointed out that the first texts related to nurses and nursing were written by Dr Ivo Mašek-Bosnodolski, as early as 1882. Dr Mašek published a book called *Dragovoljna bolesnička njega u ratu* (Voluntary Military Health Care Service), in which, among other things, he wrote about voluntary health care service and the required characteristics in women administering health care (1). The book was originally written in German and published in Zagreb, before the foundation of one of the first schools of nursing in Europe, Rudolfinerhaus in Vienna.

In the introduction of his book, Dr Mašek gave a brief description of the desirable characteristics of a nurse – kindness, warmth, subtlety, compassion, courage, and confidence in administering health care, as well as beneficial effects they have on their environment. But above all, he emphasized the need for an educated person who could provide professional help. Also, within a separate chapter, he explained in detail the necessary conditions for admission into health care service: age between 20 and 45, good health, including the health of all five senses. He pointed out the importance of the character traits, such as modesty, meekness, obedience, religiosity, humbleness, cleanliness, reliability, honesty, and probity. For the first time in the region, a special virtue was mentioned – discretion, necessary for keeping all the “secrets” important to the patient (1). At the same time, Prof Antun Lobmayer, PhD published a book called *Dvorba bolestnika* (Attending to Patients), in which he emphasized the importance of the spirit and morality of the persons caring for the sick and dying (1).

However, systematic nursing education in Croatia had not existed until 1921. That year, on January 16th, the School for Nurse-assistants, the first of that kind in the former Kingdom of Serbs, Croats, and Slovenes opened in Zagreb. Its primary aim was to professionally train nurses for anti-tuberculosis service and clinical care. The head of the school, according to regulations of the National Health Ministry Health Department in Zagreb, was a tuberculosis specialist physician. Only in 1934 was the nurse Danica Zelenjak appointed Head (2). At that time, the majority of graduated nurses worked as health teachers, social workers, and as specialist nursing course teachers. As nurses-teachers, they prepared written texts by taking notes in lectures, and these texts became the first internal course books.

## The history of the development of nursing journals

**Figure 1:**
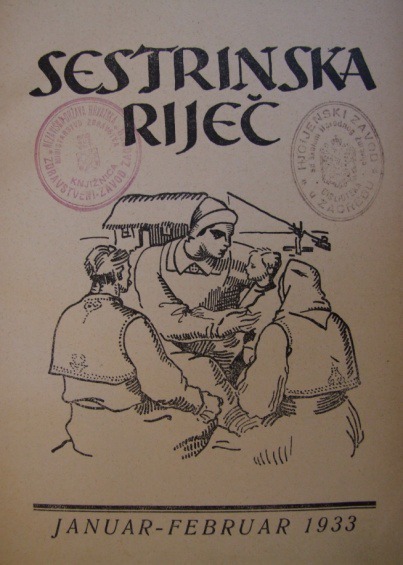
The first professional nursing publication in Croatia, specialized nursing journal *Sestrinska riječ,* 1933

Nurses started publishing their first professional publications in Croatia in a specialized nursing journal called *Sestrinska riječ* (The Word of Nurses) (3). The journal was published as a double issue, and the first issue appeared on February 1, 1933 (Figure 1).

The executive editor was nurse Lujza Janović Wagner. The journal was printed by the School of Public Health in Zagreb, and the publisher was the Association of Graduate Assistant Nurses of Yugoslavia – Section Zagreb.

The journal featured a variety of texts, and those that referred to the nursing ethics, with their clear and concise formulations, can be quoted today equally as over seventy years ago. The articles were written in Croatian, with the abstracts both in Croatian and English.

Besides professional articles and overviews of the problems within the nursing profession, the journal featured a number of translated specialized papers written by nurses abroad, which strengthened the international relations with colleagues from other countries. The journal *Sestrinska riječ* also had an impact on forming a positive public opinion on nursing as a vocation, and building up a reputation for nursing unparalleled even today. The publishing of the journal was interrupted by the World War II in 1941.

**Figure 2:**
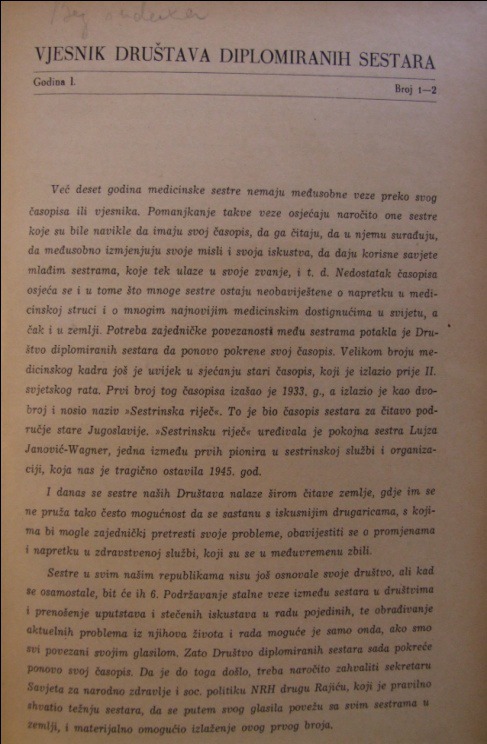
The first issue of the *Vjesnik društva diplomiranih sestara* from 1954

After the war, several vocational nursing schools were founded, but already in the early 1950s, the education for nurses was raised to a higher level. Dr Andrija Štampar, the principal of the School of Public Health and the Dean of the School of Medicine, University of Zagreb, changed the basic concept of the education of nurses. It became clear that nurses were not just doctors' assistants, subordinate to them; rather they were equally important medical workers with specific tasks. In 1950, the School of Public Health in Zagreb organized a postgraduate training and specialization for nurses. The duration of the training was three semesters and upon its completion nurses obtained a public health care nursing diploma. The courses were designed to raise the competence level within the area of general and specific patient care, dietetics, community nursing service, as well as to qualify nurses for teaching and working in public health. In 1953, the School of Medicine, University of Zagreb affiliated the School for Nurses in Zagreb, which continued its activities as the Faculty for Nurses. In this period, the idea of publishing a nursing journal re-emerged, and so, as early as 1954, the first issue of the *Vjesnik društva diplomiranih sestara* (Herald of the Graduate Nurses Associations) was published (Figure 2). The publisher was the Graduate Nurses Society of the People's Republic of Croatia, Zagreb, and the editor in chief was the nurse Ana Fajdić. The journal was published as a double issue. The great need for a specialized journal of nursing was expressed already in the introduction of the first issue:

“For the past ten years, nurses didn't have a chance of being interconnected through their own journal or herald. The lack of such communication is felt particularly by those nurses who are used to having their own magazine, reading it, being able to collaborate through it, exchange their thoughts and experiences, give useful advice to younger nurses at the beginning of their career, etc. The lack of such magazine is also felt in that many nurses remain insufficiently informed on the progress of the medical profession and on the latest medical achievements worldwide, and even in their own country.''

**Figure 3:**
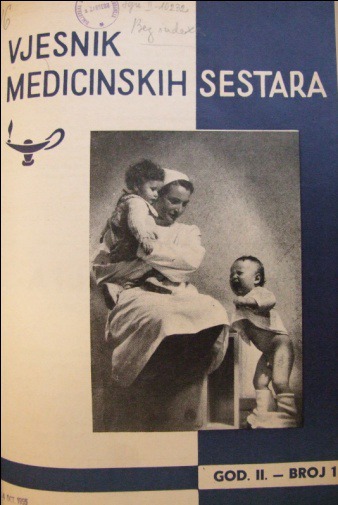
*Vjesnik medicinskih sestara,* magazine of the Association of Nurses Societies of Yugoslavia, published from 1955-1961

The interest shown by nurses was not particularly great, so that all the received articles were published. In 1955, the name of the magazine was changed into *Vjesnik medicinskih sestara* (Nursing Herald), and it was published under that name until 1961. The publisher was the Association of Nurses Societies of Yugoslavia, and Zagreb remained its place of publication. The editor in chief was Neta Dumenčić (Figure 3).

The problem of nurse’s feeble interest for writing and reading was highlighted in the introduction to the first issue:

“One should not be overly optimistic and it is illusory to think that the consciousness of the members of our professions is at a such a high level, that every third, or every fourth nurse in the country can be counted as a subscriber to the magazine.''

In the 1960s the development of nursing in Croatia was stifled; higher education for nurses was abolished and nursing was once again reduced to the level of a vocational high school, with the emphasis on acquiring technical skills. Only ten year later, there appeared a renewed possibility of education of nurses at a higher level, and with it, a renewed idea of publishing a nursing journal. Its first issue was published in 1969 under the name *Vjesnik Društva medicinskih sestara i tehničara Hrvatske* (Herald of the Society of Nurses and Medical Technicians of Croatia), and its executive editor was Ankica Pišpek. The publisher was the Society of Nurses and Medical Technicians of the People's Republic of Croatia in collaboration with the Institute for Research and Prevention of Alcoholism. In the period from 1974-1990, the journal's office was in Rijeka. It was published every three months under the name *Vjesnik medicinskih sestara i medicinskih tehničara Hrvatske* (Herald of Nurses and Medical Technicians of Croatia), and its publisher was the Association of Nurses Societies of Croatia. The editors were many – from 1977 the editor in chief was Milka Rogić, from 1981 Rozika Čuka, and from 1986 Mira Longino. Since 1979, the journal was published every two months, and its last issue No. 3-4 was published at the end of 1990.

When Croatian nurses separated from the Association of Nurses in Yugoslavia in 1992, the Croatian Nursing Association (CNA) was founded. A great number of nurses and medical technicians actively participated in the Croatian War of Independence, providing health care for soldiers at the battlefield or for those in medical institutions. The majority of professional publications written by nurses were published in various handbooks, which served as manuals for self care or attending to the wounded, or as manuals for social or psychological support. As early as 1995, the Croatian Nursing Association started publishing its magazine called *Sestrinski glasnik* (Nursing Herald). In the beginning, it featured reportage and interviews with people who significantly contributed to the development of Croatian nursing and Croatian society, while in the last five years the magazine has focused mostly on publishing international professional publications in the field of nursing.

After its founding, the Croatian Nursing Council began publishing a journal called *Plavi fokus* (Blue Focus). Blue Focus publishes news related to the Council's activities, reports on events related to nursing profession in Croatia and worldwide, interviews, and professional publications in the field of health care.

## History of publishing course books and manuals in the nursing care field

Nearly forty years passed from the founding of the first school for nurses until publishing of the first course book adapted for acquiring knowledge related to the nursing care field. It should be pointed out that in the 1960s these course books were mostly prepared and written by doctors. Such course books surely had a great value; however, their main disadvantage was ''the doctor's viewpoint'' of the nursing practice. Until the 1990s, only two course books written independently by nurses were published (Table 1).

**Table 1:** Coursebooks from the nursing care field in the 1950s and 1960s

Author(s) – doctors	Coursebook title	Publisher and place and year of publication
Svebor Čerlek	Internal Medicine for Nurses with Special Care, Medical Technique and Diet for Patients (*Interna medicina za sestre sa specijalnom njegom, medicinskom tehnikom i prehranom bolesnika*)	Institute for Health Protection, 1958
Predrag Drobnjak, Eduard Barišić	Gynecology and Obstetrics (*Ginekologija i porodiljstvo*)	Medical Book, Beograd-Zagreb, 1964
Boško Milojević	Otorynolaringology (*Otorinolaringologija*)	Medical Book, Beograd-Zagreb, 1964
Josip Fališevac	Infectious Diseases: Etiology, Epidemiology, Clinics, Prophylactics (*Zarazne bolesti: etiologija, epidemiologija, klinika, profilaksa*)	Medical Book, Beograd-Zagreb, 1964
Ivan Prpić	Surgery (*Kirurgija*)	Medical Book, Beograd-Zagreb, 1964
Vlado Ilić	Hare Care Education: Methods, Means, and Specific Tasks	Institute for Health Protection, 1967

Since Croatia gained independence and introduced new higher education programs for nurses, we have witnessed the flourishing of nursing course books, whose authors, among other things, had obtained nursing education and relevant experience in the nursing practice. And while the 1990s saw the publishing of only two course books in Croatian: *Proces zdravstvene njege* (Health Care Process) (4) and *Sestrinska dijagnoza (*Nursing Diagnosis) (5), in the first decade of the 21st century, nurses published more than 10 course books covering various areas of health care.

And finally, this served as a motivation to consider the need for a scientific journal of nursing, which would be published in Croatian and which would enable nurses to publish professional and scientific publications, necessary for spreading knowledge, but also for obtaining references for professional and scientific advances within the profession.
